# A Significantly Higher Glucose Concentration in Plasma Collected with Glycolytic Inhibitors than in Serum: Impact of Insulin Resistance

**DOI:** 10.3390/nu18050813

**Published:** 2026-03-02

**Authors:** Akihiro Yoshida, Takumi Nagasawa, Madoka Inoue, Suguru Hiramoto, Fumitaka Murakami, Mari Hashimoto, Sakura Motoki, Mayumi Nishiyama, Katsuhiko Tsunekawa, Takao Kimura

**Affiliations:** 1 Department of Clinical Laboratory Medicine, Gunma University Graduate School of Medicine, Showa-Machi 3-39-22, Maebashi 371-8511, Gunma, Japan; ayossie10@gunma-u.ac.jp (A.Y.); t_nagasawa@gunma-u.ac.jp (T.N.); madoka_4_4@gunma-u.ac.jp (M.I.); s-hiramoto@gunma-u.ac.jp (S.H.); f2510044@gunma-u.ac.jp (S.M.); mayumin@gunma-u.ac.jp (M.N.); ktsune@gunma-u.ac.jp (K.T.); 2 Clinical Laboratory Center, Gunma University Hospital, Showa-Machi 3-39-22, Maebashi 371-8511, Gunma, Japan; f.murakami.1202@gunma-u.ac.jp (F.M.); mari.ha@gunma-u.ac.jp (M.H.); 3 Department of Medical Education and Development, Gunma University Graduate School of Medicine, Showa-Machi 3-39-22, Maebashi 371-8511, Gunma, Japan; 4 Department of Integrated Health Science, Graduate School of Food and Population Health Science, Gunma University, Aramaki-Machi 4-2, Maebashi 371-8510, Gunma, Japan; 5 Ikuei University, Kyome-Machi 1656-1, Takasaki 370-0011, Gunma, Japan; 6 General Health Support Center, Gunma University, Aramaki-Machi 4-2, Maebashi 371-8510, Gunma, Japan

**Keywords:** serum, plasma, glucose concentration, glycolysis, insulin resistance, sodium fluoride–citrate, high-density lipoprotein cholesterol

## Abstract

**Objectives**: This study aimed to identify factors influencing the magnitude of the difference between plasma glucose concentration (Glu(P)) and serum glucose concentration (Glu(S)). **Methods**: A total of 333 healthy Japanese adults aged 22–29 years (212 males and 121 females) were enrolled. Plasma samples were collected using glycolytic inhibitors, whereas serum samples were obtained without glycolytic inhibitors and kept at room temperature. Glu(P) and Glu(S) were measured and compared. **Results**: The median difference between Glu(P) and Glu(S), defined as Glu(P-S), was 4 mg/dL across all participants, with no gender-related differences. A strong positive correlation was observed between Glu(P) and Glu(S). Glu(P-S) was positively correlated with body mass index, Glu(P), triglyceride–glucose index, white blood cell count, serum sodium, magnesium, and zinc levels. In contrast, Glu(P-S) was negatively correlated with Glu(S), hemoglobin A1c (HbA1c), homeostasis model assessment of beta-cell function, and high-density lipoprotein cholesterol (HDL-C). Multiple regression analysis demonstrated that HDL-C and HbA1c were independent determinants of Glu(P-S) in the overall cohort. Among females, HDL-C, triglyceride, low-density lipoprotein cholesterol, ferritin, and *C*-reactive protein independently influenced Glu(P-S), whereas no independent determinants were identified in males. **Conclusions**: Plasma glucose concentrations measured with glycolytic inhibitors were significantly higher than serum glucose concentrations measured without inhibitors at room temperature. The magnitude of Glu(P-S) appears to be associated with markers of insulin resistance, particularly HDL-C levels.

## 1. Introduction

Effective screening for impaired fasting glucose, impaired glucose tolerance, prediabetes, and diabetes requires accurate and cost-effective measurement of glucose in peripheral blood, ideally together with hemoglobin A1c (HbA1c), from a single blood draw. Current guidelines for the diagnosis and management of diabetes mellitus recommend measuring glucose in venous plasma and minimizing glycolysis by immediately placing blood collection tubes in an ice-water slurry or by using citrate-buffered tubes [1]. Nevertheless, several studies have assessed glucose tolerance using serum glucose measurements [2,3,4,5]. In low- to middle-income countries in particular, substantial cost savings could be achieved if glucose tolerance could be reliably evaluated using serum glucose levels. Recently, Bakkebø et al. demonstrated that the plasma glucose concentrations measured at room temperature in samples collected with a sodium fluoride (NaF)–citrate mixture, an effective glycolytic inhibitor, were significantly higher than glucose concentrations measured in plasma or serum collected under ice-cooling conditions without NaF–citrate [6]. For accurate assessment of glucose tolerance, measurement of plasma glucose using NaF–citrate is therefore considered the optimal approach. However, when serum glucose concentrations are used in a clinical or epidemiological setting, it is important to clarify the factors that influence their accuracy.

During blood sample processing, glycolysis proceeds within erythrocytes through a pathway involving 13 enzymes [7]. Citrate rapidly inhibits 6-phosphofructokinase, an upstream regulatory enzyme in glycolysis, within approximately 4 min [8,9]. Sodium fluoride inhibits glycolysis by binding to enolase (Eno1) and pyruvate kinase in erythrocytes [10]. Eno1 is a multifunctional protein expressed in various cell types and tissues and has recently gained attention for its potential role in the pathogenesis of diabetes. Experimental studies have shown associations between Eno1 activity and insulin secretion as well as insulin sensitivity and resistance [11,12,13,14].

These findings suggest that the difference between glucose concentration measured in plasma collected with NaF–citrate (Glu(P)) and in serum collected without NaF–citrate (Glu(S)), hereafter referred to as Glu(P-S)), may partially reflect glycolytic activity within erythrocytes. However, the relationship between erythrocyte glycolytic activity and insulin secretion or insulin sensitivity/resistance in humans remains unclear. Therefore, the objectives of this study were twofold: first, to quantify the magnitude of Glu(P-S) and, second, to identify factors associated with variations in Glu(P-S).

## 2. Methods

The study was conducted in accordance with the Declaration of Helsinki and approved by the ethical board of Gunma University Hospital (HS2024-220, approved on 25 November 2024). This study was conducted using an opt-out method, with a public notice posted on the hospital’s website. Each participant was given the opportunity to decline participation. We reanalyzed data from a previous study [15]. Participants in this study were those who had data on both plasma and serum glucose concentrations and body fat percentage from previous studies [15]. The study period was from June 2012 to July 2016. The study cohort comprised 333 healthy young Japanese adults aged 22–29 years (212 males and 121 females). Blood samples were collected between 9:00 and 9:30 am. Subjects were required to fast for 10 h before the test, but there were no restrictions on exercise. Weight and body composition (body fat percentage and muscle mass) were measured using the INBODY470 system (InBody Japan Inc., Tokyo, Japan).

Blood samples were collected into the following tubes: rapid serum tubes (INSEPACK II-DSMD760CG, Sekisui, Tokyo, Japan) containing thrombin as a clotting activator for rapid serum separation, VENOJECT II tubes (VP-FH052K; NIPRO, Osaka, Japan) containing citrate and NaF for plasma glucose and HbA1c measurements, and NEO-TUBE (ANP-EK0205; NIPRO, Osaka, Japan) for the complete blood count.

**Figure 1:**
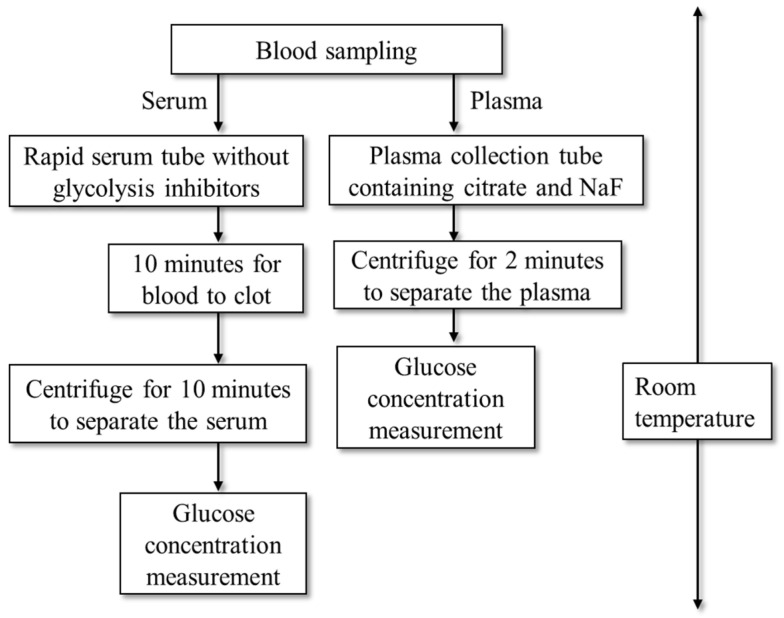
Workflow from blood collection to measurement of plasma and serum glucose concentrations at the Clinical Laboratory Center, Gunma University Hospital. RST, rapid serum tube; NaF, sodium fluoride.

Fasting plasma glucose and HbA1c were measured immediately after blood collection using the ADAMS Glucose GA-1170 and ADAMS A1c HA8182 analyzers (Arkray, Tokyo, Japan). Complete blood count parameters—including white blood cell count (WBC), hemoglobin (Hb), and platelet count—were analyzed using an XE-5000 hematology system (Sysmex, Kobe, Japan). Serum samples for clinical chemistry were centrifuged 10 min after collection, and the following parameters were measured using the LABOSPECT008α analyzer (Hitachi, Tokyo, Japan): serum glucose, insulin, high-density lipoprotein cholesterol (HDL-C), low-density lipoprotein cholesterol (LDL-C), triglycerides (TGs), γ-glutamyltransferase (γGT), total protein, albumin, sodium (Na), potassium, chlorine, calcium, inorganic phosphorus, iron (Fe), ferritin, magnesium (Mg), and zinc (Zn). All blood collection tubes were handled at room temperature (approximately 20 °C). A schematic of the workflow from blood collection to reporting of plasma and serum glucose levels at the Department of Clinical Laboratory Medicine, Gunma University, is shown in Figure 1. The elapsed time from blood sampling to glucose result reporting was at least 20 min for serum and 4 min for plasma.

Insulin resistance was assessed using the homeostasis model assessment for insulin resistance (HOMA-IR), calculated as fasting plasma glucose (PG_0_, mg/dL) × fasting immunoreactive insulin (IRI_0_, μU/mL)/405 [16]. Pancreatic β-cell function was evaluated using homeostasis model assessment of β-cell function (HOMA-β), calculated as IRI_0_ (μU/mL) × 360/[PG_0_ (mg/dL) − 63] [16]. The triglyceride–glucose (TyG) index, an indicator of insulin resistance, was calculated as ln[fasting TG (mg/dL) × fasting glucose (mg/dL)/2] [17].

All statistical analyses were performed using the Statistical Package for the Social Sciences version 30. Glu(P) and Glu(S) were compared using the Wilcoxon signed-rank test for paired samples. Agreement between plasma and serum glucose was assessed by Bland–Altman analysis, and a linear regression model was created for Glu(P) and Glu(S). Correlations between Glu (P-S) and other parameters were analyzed using Spearman’s rank correlation coefficient. Comparison of parameters between groups with positive versus negative Glu(P-S) values was performed using the Mann–Whitney U-test, stratified by gender. Multiple regression analysis was conducted with Glu(P-S) as the dependent variable, both overall and by gender.

## 3. Results

Table 1 summarizes the characteristics of the 333 healthy young Japanese participants. Given known gender differences in glucose tolerance [15,18,19,20], data are presented for all participants, as well as separately for males and females. Compared with females, males had significantly higher age, body mass index (BMI), Glu(P), Glu(S), TG, HOMA-IR, TyG index, γGT, Hb, ferritin, and serum Fe. In contrast, body fat percentage, HbA1c, HDL-C, HOMA-β, and platelet count were lower in males than in females. The median Glu(P-S) was 4 mg/dL across all participants, with no significant difference between males and females (Table 1 and Supplemental Figure S1). These findings suggest that among young, healthy Japanese adults, females exhibit greater insulin sensitivity and higher basal beta-cell function than males.

**Figure 2:**
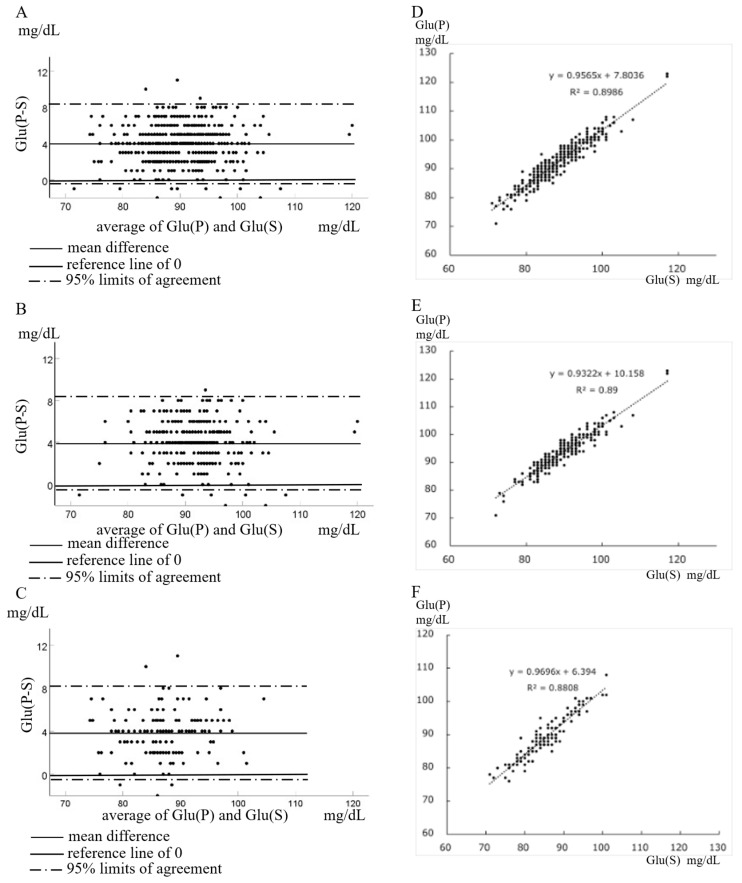
Bland–Altman analysis of plasma and serum glucose (**A**–**C**) and linear regression between plasma and serum glucose (**D**–**F**). (**A**) All participants (*n* = 333), (**B**) males (*n* = 212), (**C**) females (*n* = 121). (**D**) All participants (*n* = 333), (**E**) males (*n* = 212), (**F**) females (*n* = 121). Regression equations and correlation coefficients are shown.

As shown in Table 1 and Figure 2, Glu(P) was significantly higher than Glu(S) in all participants, irrespective of gender. Notably, a small number of subjects had higher Glu(S) than Glu(P)—7 males and 3 females. Bland–Altman analysis comparing Glu(P) and Glu(S) is presented in Figure 2A–C (Figure 2A: all participants, Figure 2B: males, and Figure 2C: females). The mean magnitude of Glu(P-S) was 3.96 mg/dL overall, 4.06 mg/dL in males, and 3.802 mg/dL in females. Linear regression analyses of Glu(S) versus Glu(P) are shown in Figure 2D–F (Figure 2D: all participants, Figure 2E: males, and Figure 2F: females), demonstrating a strong positive correlation between plasma and serum glucose levels in each group.

Simple correlation analyses between Glu(P-S) and clinical or laboratory parameters are summarized in Table 2. In the overall cohort, Glu(P-S) was positively correlated with BMI, Glu(P), TyG index, WBC, Na, Mg, and Zn, and negatively correlated with Glu(S), HbA1c, HOMA-β, and HDL-C. Among males, Glu(P-S) was positively correlated with TG, TyG index, WBC, and Na and negatively correlated with Glu(P) and HbA1c. Among females, Glu(P-S) was positively correlated with Glu(P) and Mg and negatively correlated with HOMA-β and HDL-C. Correlations between plasma and serum glucose and other metabolic parameters are shown in Table 3 and Supplemental Table S1A–C.

In the overall cohort, both Glu(P) and Glu(S) were positively correlated with insulin, HOMA-IR, TyG index, red blood cell count, Hb, and ferritin, and negatively correlated with HDL-C. Glu(P) was negatively correlated with HOMA-β, whereas Glu(S) was positively correlated with HbA1c (Table 3 and Supplemental Table S1A). Among males, Glu(P) and Glu(S) were positively correlated with insulin and HOMA-IR, but only Glu(P) showed positive correlations with the TyG index and WBC (Table 3, Supplemental Table S1B). Among females, both Glu(P) and Glu(S) were positively correlated with insulin, HOMA-IR, and the TyG index, while only Glu(P) showed a positive correlation with ferritin (Table 3 and Supplemental Table S1C).

A subset of participants exhibited higher Glu(S) than Glu(P). Among males, 197 had positive Glu(P-S), 5 had equivalent Glu(P) and Glu(S), and 7 had negative Glu(P-S). Among females, 113, 6, and 3 participants fell into these categories, respectively. To further investigate this, Welch’s *t*-test was performed, comparing the groups with higher Glu(P) versus those with higher Glu(S) (Supplemental Table S2). In males with negative Glu(P-S), Glu(S) was significantly higher, and serum Fe was lower.

Multiple regression analysis was performed with Glu(P-S) as the dependent variable (Table 4). Candidates for independent variables included age, BMI, body fat, HbA1c, hemoglobin, WBC, platelet count, insulin, HDL-C, TG, LDL-C, Na, potassium, chlorine, Mg, Fe, ferritin, Zn, and γGT. In the overall cohort, HDL-C and HbA1c were independent negative predictors of Glu(P-S), indicating that Glu(P-S) decreases with higher HbA1c and HDL-C levels. In females, Glu(P-S) decreased with increasing HDL-C and TG but increased with higher LDL-C, ferritin, and *C*-reactive protein. No independent predictors were identified in males.

## 4. Discussion

In this study, plasma glucose concentrations measured using glycolytic inhibitors at room temperature were significantly higher than serum glucose concentrations measured without inhibitors in healthy young Japanese adults. The median Glu(P-S) was approximately 4 mg/dL across all participants, with no significant gender difference. Multiple regression analysis indicated that the magnitude of Glu(P-S) is associated with insulin sensitivity/resistance, particularly HDL-C. These results suggest that using serum glucose measurements to assess glucose tolerance may underestimate glucose levels in individuals with higher insulin resistance.

Glu(P-S) was positively correlated with the TyG index, a marker of insulin resistance [16,21], in the overall cohort and in males. Conversely, in females and the overall cohort, Glu(P-S) was negatively correlated with HOMA-β and HDL-C, while TG was positively correlated with Glu(P-S) in males. Circulating HDL-C, TG, and HOMA-β were also significantly correlated with HOMA-IR and the TyG index in this study population, consistent with previous reports [15,18,19,20,22,23]. HDL-C, LDL-C, TG, Hb, Fe, ferritin, and γGT are not affected by insulin resistance during the blood sampling and measurement process. Collectively, these findings suggest that under room temperature conditions, individuals with higher insulin resistance exhibit a greater Glu(P-S). Therefore, plasma glucose measured with glycolytic inhibitors is recommended when assessing glucose tolerance in overweight or obese individuals or those suspected of impaired fasting glucose, impaired glucose tolerance, or diabetes. Reliance on serum glucose alone at room temperature may lead to underestimation of glucose tolerance.

The participants in this study had physiologically normal characteristics. Consistent with prior studies [15,18,19,20,22], females had higher body fat, HOMA-β, HbA1c, HDL-C, and platelet counts, whereas males had higher fasting plasma glucose, fasting insulin, HOMA-IR, TyG index, TG, Hb, Fe, ferritin, and γGT. Glu(S) was positively correlated with HbA1c in all participants and in males, but not in females, whereas Glu(P) showed no correlation with HbA1c. This may reflect low glycolytic activity in erythrocytes, resulting in relatively high HbA1c despite normal glucose tolerance—a hypothesis requiring further investigation. Gender differences in basal insulin secretion and insulin sensitivity were also reflected in correlations between Glu(S) and the TyG index. The females in this study had significantly lower Fe and ferritin levels than males due to menstruation. Among females, low Fe was related to increased HbA1c levels, consistent with a previous report [24]. It is thought that low Fe and ferritin levels led to relatively high HbA1c levels in females. Among males, LDL-C is associated with BMI, HbA1c, insulin, HOMA-IR, HOMA-β, TG, and TyG index, while LDL-C is associated with TG and TyG index among females. Compared to females, males had higher Glu(P), Glu(S), HOMA-IR, TyG index, and TG, and lower HOMA-β and HDL-C. These facts are thought to be some of the reasons why a significant correlation was observed between Glu(S) and HbA1c in males, but not in females. These differences may explain why no independent predictors of Glu(P-S) were identified in males in multiple regression analysis. When multiple regression analysis was performed for males using the elimination method, HbA1c and TG remained at the bottom with a significance probability of less than 0.1. If the number of cases had been larger, it may have been possible to create a regression equation. In contrast, HDL-C and HbA1c were independent predictors in the overall cohort, and HDL-C, TG, LDL-C, ferritin, and *C*-reactive protein were independent predictors in females, despite no gender difference in the magnitude of Glu(P-S).

Glycolysis in erythrocytes is mediated by a 13-enzyme pathway [7]. NaF inhibits glycolysis by binding to Eno1 [10], a multifunctional protein also involved in insulin secretion and resistance [11,12,13,14]. These findings suggest that Eno1 expression and activity in erythrocytes influence glucose reduction during serum separation and may be modulated by insulin sensitivity/resistance. Enolase activity depends on divalent metal ions, including Mg, manganese, Zn, and cobalt [25], which is consistent with the observed associations between Glu(P-S) and serum Mg and Zn. Additionally, glycolysis-generated ATP fuels Na^+^-K^+^-ATPase, which maintains erythrocyte and serum sodium homeostasis [26], aligning with the positive correlation between Glu(P-S) and serum Na observed in this study.

For accurate glucose measurement at room temperature, plasma should be collected using glycolytic inhibitors such as citrate, which inhibits glycolysis more effectively than NaF for 4 min to 4 h post-collection [9,27]. Bakkebø et al. showed that glucose in plasma collected with NaF–citrate at room temperature was higher than in plasma collected using lithium-heparin with immediate cooling and higher than in serum [6]. These data suggest that inhibitor presence may be more critical than temperature control. However, if the use of glycolysis inhibitors such as citrate is difficult due to cost or analytical environment constraints, another option is to measure blood glucose levels by cooling the blood on ice immediately after collection [1]. Nevertheless, a few participants in this study had higher serum glucose than plasma glucose, which may reflect interference during serum clotting, hemolysis, and matrix effects; the precise mechanism remains unclear.

## 5. Limitations and Future Perspectives

This study has several limitations. First, it was conducted at a single institution, which may limit generalizability. Multicenter studies are needed to validate these findings. Second, the study population consisted of only healthy young Japanese adults, restricting extrapolation to other ages, ethnicities, or individuals with a chronic illness; future studies in broader and diverse cohorts are required. Third, variations in blood collection tubes, analyzers, and storage conditions across institutions could affect reproducibility. Fourth, guideline-compliant blood collection using dedicated tubes may be cost-prohibitive in some settings; however, understanding deviations from standard methods remains valuable. Fifth, Eno1 expression and activity were not measured, leaving the mechanistic link between glycolysis, metal ions, and Glu(P-S) speculative. Analysis of Eno1 activity in erythrocytes will address this issue. Sixth, there were many parameters for which relatively weak correlations were observed. This may have been due to the relatively small sample size. Finally, while regression analyses suggested associations between Glu(P-S) and markers of glucose–lipid metabolism, underlying mechanisms, particularly regarding insulin resistance, remain to be elucidated. Despite these limitations, the study provides a foundation for future large-scale, multicenter investigations.

## 6. Conclusions

Under room-temperature conditions, plasma glucose measured with glycolytic inhibitors is significantly higher than serum glucose. The activity of erythrocyte glycolysis, influenced by insulin sensitivity/resistance and electrolyte concentrations, affects the magnitude of Glu(P-S). Accordingly, glucose tolerance assessments based solely on serum glucose carry a risk of underestimation, highlighting the importance of using plasma collected with glycolytic inhibitors for accurate evaluation. If it is difficult to use glycolysis inhibitors such as citrate, the blood should be immediately cooled on ice after collection, and the serum should be separated for glucose measurement.

## Figures and Tables

**Table 1:** Clinical characteristics of participants.

	All Participants (*n* = 333)	Males (*n* = 212)	Females (*n* = 121)	*p*
Age (years)	24 (23−25)	24 (23−25)	23 (23−24)	<0.01 *
BMI (kg/m^2^)	21.5 (20−23.4)	22.4 (20.9−24.0)	20.2 (18.9−22)	<0.01 *
Body fat (%)	21.9 (17.5−26.7)	18.5 (15.6−22.8)	27.6 (24.5−30.4)	<0.01 *
Plasma glucose (mg/dL)	93 (88−97)	94 (90−98)	89 (85−94)	<0.01 *
Serum glucose (mg/dL)	88 (84−92)	90 (86−93)	85 (82−89)	<0.01 *
Glu(P-S)	4 (2−5)	4 (3−6)	4 (2−5)	0.312
HbA1c (%)	5.4 (5.2−5.5)	5.4 (5.2−5.5)	5.4 (5.3−5.5)	0.038 *
Insulin (μU/mL)	6.7 (5.1−9.0)	6.8 (5.4−9.2)	6.5 (4.9−8.6)	0.702
HDL-C (mg/dL)	62 (54−73)	58 (50−65)	72 (64−79)	<0.01 *
Triglyceride (mg/dL)	60 (45−83)	66 (51−90)	50 (41−67)	<0.01 *
LDL-C (mg/dL)	100 (84−119)	101 (86−121)	98 (81−116)	0.188
HOMA-IR	1.51 (1.12−2.10)	1.56 (1.20−2.23)	1.38 (1.01−1.94)	0.025 *
HOMA-β	84.0 (64.4−110.2)	80.7 (63.4−106.2)	90.9 (74.5−119.3)	0.021 *
TyG index	7.92 (7.62−8.27)	8.05 (7.78−8.35)	7.73 (7.52−7.99)	<0.01 *
γGT (mg/dL)	17 (13−24)	20 (15−26)	13 (11−18)	<0.01 *
Hb (g/dL)	15.0 (13.9−15.9)	15.6 (15.1−16.2)	13.6 (12.9−14)	<0.01 *
WBC (×10^3^/μL)	5.0 (4.3−5.8)	4.9 (4.3−5.7)	5.1 (4.4−6.0)	0.327
Plt (×10^3^/μL)	239 (211−268)	229 (206−260)	257 (224−277)	<0.01 *
Fe (mg/dL)	118 (89−147)	122 (90−151)	108 (85−143)	0.037 *
Ferritin (ng/mL)	79.1 (34.8−161.8)	136.1 (86.1−195.4)	31.1 (16.5−48.4)	<0.01 *

Data are shown as medians (first quartile–third quartile). BMI: Body Mass Index; Glu(P-S): plasma glucose-serum glucose; HbA1c: hemoglobin A1c; IRI: immunoreactive insulin; HDL-C: high-density lipoprotein cholesterol; TG: triglyceride; LDL-C: low-density lipoprotein cholesterol; TyG index: Triglyceride Glucose index; γGT: γ-glutamyltransferase; Hb: hemoglobin; WBC: white blood cell count; Plt: platelet count; Fe: iron. Significance between-groups differences were identified using the Mann–Whitney U test. Statistical significance was set at a *p*-value of <0.05. *: *p* < 0.05. Male > female in bold red, Male < female in bold blue.

**Table 2:** Correlation analysis of Glu(P-S) and each parameter.

	All Participants (*n* = 333)		Males (*n* = 212)		Females (*n* = 121)	
	*r*	*p*	*r*	*p*	*r*	*p*
Age	−0.053	0.334	−0.119	0.085	0.033	0.718
BMI	0.132	0.017 *	0.1	0.148	0.117	0.207
Body fat	−0.034	0.539	0.056	0.415	−0.011	0.902
Glu(P)	0.193	<0.01 *	0.115	0.096	0.275	0.002 *
Glu(S)	−0.124	0.024 *	−0.228	<0.01 *	−0.086	0.35
HbA1c	−0.138	0.012 *	−0.229	<0.01 *	0.039	0.668
Insulin	−0.036	0.515	−0.015	0.834	−0.09	0.327
HOMA-IR	−0.011	0.846	0	0.996	−0.047	0.612
HOMA-β	−0.165	0.003 *	−0.085	0.219	−0.284	<0.01 *
HDL-C	−0.109	0.047 *	−0.035	0.612	−0.238	<0.01 *
TG	0.098	0.074	0.141	0.040 *	−0.018	0.842
LDL-C	−0.017	0.751	−0.059	0.392	0.054	0.559
TyG index	0.122	0.026 *	0.155	0.024 *	0.023	0.799
γGT	0.107	0.051	0.123	0.074	0.047	0.609
Hb	0.072	0.187	0.02	0.776	0.011	0.904
WBC	0.153	0.005 *	0.164	0.017 *	0.154	0.092
Plt	0.022	0.69	0.017	0.801	0.057	0.532
Na	0.146	<0.01 *	0.175	0.011 *	0.061	0.504
K	0.059	0.28	0.026	0.703	0.081	0.376
Cl	−0.01	0.851	0.023	0.743	−0.046	0.615
IP	−0.026	0.656	−0.102	0.158	0.131	0.177
Ca	−0.068	0.229	−0.096	0.17	−0.069	0.472
Mg	0.128	0.025 *	0.06	0.409	0.249	<0.01 *
Fe	0.055	0.317	0.089	0.198	−0.015	0.872
Ferritin	0.136	0.076	0.058	0.558	0.155	0.206
Zn	0.245	0.016 *	0.149	0.291	0.242	0.114
CRP	0.076	0.168	0.074	0.285	0.039	0.671

Data are shown as Spearman’s rank correlation coefficient for each parameter. BMI: Body Mass Index; HbA1c: hemoglobin A1c; Glu(P): plasma glucose; Glu(S): serum glucose; IRI: immunoreactive insulin; HOMA-IR: Homeostatic Model Assessment for Insulin Resistance; HOMA-β: Homeostatic Model Assessment of beta cell function; HDL-C: high-density lipoprotein cholesterol; TG: triglyceride; LDL-C: low-density lipoprotein cholesterol; TyG index: Triglyceride Glucose index; γGT: γ-glutamyltransferase; Hb: hemoglobin; WBC: white blood cell count; Plt: platelet count; Na: sodium; K: potassium; Cl: chlorine; IP: inorganic phosphorus; Ca: calcium; Mg: magnesium; Fe: iron; Zn: zinc; CRP: C-reactive protein. Statistical significance was set at a *p*-value of <0.05. *: *p* < 0.05. Positive correlations are in bold red, negative correlations are in bold blue.

**Table 3:** Representative data of simple correlation analysis between glucose concentration and other parameteres.

			Glu(P)	Glu(S)	HbA1c	IRI	HOMA-IR	HOMA-b	HDL-C	TG	LDL-C	TyG Index	RBC	Hb	WBC	Ferritin	Fe	Na	Mg	Zn
All participants *n* = 313	BMI	*r*	0.252	0.206	−0.063	0.171	0.196	0.074	−0.358	0.194	0.219	0.218	0.382	0.391	0.119	0.558	0.077	0.1	0.067	0.229
		*p*	**<** **0** .001 *	**<** **0** .001 *	0.256	0.002 *	**<** **0** .001 *	0.179	**<** **0** **.001**	**<** **0** .001 *	**<** **0** .001 *	**<** **0** .001 *	**<** **0** .001 *	**<** **0** .001 *	0.031 *	**<** **0** .001 *	0.162	0.07	0.247	0.025 *
	Glu(P)	*r*		0.938	0.079	0.403	0.517	−0.135	−0.235	0.143	0.076	0.284	0.283	0.302	0.096	0.335	0.025	0.064	0.069	0.105
		*p*		**<** **0** .001 *	0.148	**<** **0** .001 *	**<** **0** .001 *	0.014 *	**<** **0** .001 *	0.009 *	0.167	**<** **0** .001 *	**<** **0** .001 *	**<** **0** .001 *	0.081	**<** **0** .001 *	0.649	0.242	0.232	0.309
	Glu(S)	*r*			0.133	0.428	0.535	-0.083	−0.209	0.107	0.078	0.242	0.262	0.274	0.046	0.295	-0.002	0.023	0.034	0.02
		*p*			0.015 *	**<** **0** .001 *	**<** **0** .001 *	0.132	**<** **0** .001 *	0.052	0.157	**<** **0** .001 *	**<** **0** .001 *	**<** **0** .001 *	0.402	**<** **0** .001 *	0.967	0.676	0.552	0.844
Males *n* = 212	BMI	*r*	0.119	0.075	0.015	0.204	0.206	0.209	−0.292	0.145	0.284	0.147	0.227	0.211	0.185	0.229	0.065	−0.051	0	0.219
		*p*	0.085	0.28	0.825	0.003 *	0.003 *	0.002 *	**<** **0** .001 *	0.035 *	**<** **0** .001 *	0.032 *	**<** **0** .001 *	0.002 *	0.007 *	0.02 *	0.349	0.461	0.999	0.119
	Glu(P)	*r*		0.925	0.134	0.394	0.502	−0.073	−0.036	0.048	0.076	0.179	0.025	0.088	0.15	−0.04	0.036	−0.06	0.082	−0.087
		*p*		**<** **0** .001 *	0.051	**<** **0** .001 *	**<** **0** .001 *	0.292	0.6	0.484	0.27	0.009 *	0.719	0.2	0.029 *	0.689	0.603	0.382	0.254	0.54
	Glu(S)	*r*			0.205	0.402	0.503	−0.038	−0.032	−0.006	0.081	0.118	0.024	0.066	0.105	−0.037	−0.01	−0.094	0.047	−0.151
		*p*			0.003 *	**<** **0** .001 *	**<** **0** .001 *	0.585	0.642	0.933	0.239	0.087	0.731	0.338	0.127	0.712	0.885	0.173	0.517	0.285
Females *n* = 121	BMI	*r*	0.16	0.109	−0.104	0.089	0.107	0.011	−0.121	0.064	0.124	0.086	−0.113	−0.15	0.13	0.387	0.013	0.049	0.109	−0.305
		*p*	0.082	0.237	0.259	0.333	0.247	0.908	0.191	0.493	0.178	0.353	0.221	0.104	0.159	0.001 *	0.892	0.594	0.262	0.047 *
	Glu(P)	*r*		0.909	0.113	0.412	0.515	−0.163	−0.13	0.1	0.046	0.272	0.027	-0.024	0.032	0.27	−0.095	0.015	−0.018	0.148
		*p*		**<** **0** .001 *	0.217	**<** **0** .001 *	**<** **0** .001 *	0.074	0.156	0.277	0.614	0.003 *	0.773	0.79	0.729	0.026 *	0.301	0.871	0.856	0.336
	Glu(S)	*r*			0.145	0.471	0.562	−0.059	−0.061	0.094	0.046	0.26	−0.017	−0.04	−0.018	0.182	−0.101	−0.026	−0.057	0.064
		*p*			0.112	**<** **0** .001 *	**<** **0** .001 *	0.523	0.509	0.304	0.613	0.004 *	0.856	0.663	0.844	0.137	0.271	0.78	0.557	0.677

Data are shown as Spearman’s rank correlation coefficient for each parameter. BMI: Body Mass Index; HbA1c: hemoglobin A1c; Glu(P): plasma glucose; Glu(S): serum glucose; IRI: immunoreactive insulin; HOMA-IR: Homeostatic Model Assessment for Insulin Resistance; HOMA-β: Homeostatic Model Assessment of beta cell function; HDL-C: high-density lipoprotein cholesterol; TG: triglyceride; LDL-C: low-density lipoprotein cholesterol; TyG index: Triglyceride Glucose index; γGT: γ-glutamyltransferase; Hb: hemoglobin; WBC: white blood cell count; Plt: platelet count; Na: sodium; K: potassium; Cl: chlorine; IP: inorganic phosphorus; Ca: calcium; Mg: magnesium; Fe: iron; Zn: zinc; CRP: C-reactive protein. Statistical significance was set at a *p*-value of <0.05. *: *p* < 0.05. Positive correlations are in bold red, negative correlations are in bold blue.

**Table 4:** Multiple regression analysis with the difference between plasma glucose and serum glucose as the dependent variable.

		Unstandardized		Standardized			95.0% Confidence Interval for B		Collinearity Statistics	
		Coefficients		Coefficients						
	Model	B	Standard Error.	Beta	t	Sig.	Lower Bound	Upper Bound	Tolerance	VIF
All participants (*n* = 333)	(Constant)	19.531	5.741		3.402	0.001	8.059	31.002		
	HDL-C	−0.053	0.017	−0.354	−3.076	0.003	−0.088	−0.019	0.988	1.013
	HbA1c	−2.431	1.029	−0.272	−2.362	0.021	−0.487	−0.374	0.988	1.013
Females (*n* = 121)	(Constant)	4.78	2.184		2.189	0.038	0.283	9.277		
	HDL-C	−0.062	0.025	−0.377	−2.437	0.022	−0.114	−0.01	0.954	1.048
	TG	−0.03	0.01	−0.574	−3.021	0.006	−0.05	−0.009	0.633	1.581
	LDL-C	0.025	0.012	0.344	2.122	0.044	0.001	0.049	0.868	1.152
	Ferritin	0.039	0.015	0.437	2.654	0.014	0.009	0.07	0.84	1.191
	CRP	11.753	5.417	0.369	2.17	0.04	0.597	22.909	0.79	1.265
Males (*n* = 212)		No significant regression equation could be constructed.								

Variables attempted to be entered: age, BMI, body fat, HbA1c, Hb, WBC, Plt, IRI, HDL-C, TG, LDL-C, Na, K, Cl, Mg, Fe, ferritin, Zn, γGT, and CRP. Excluded variables among all participants: age, BMI, body fat, Hb, WBC, Plt, IRI, Na, K, Cl, Mg, Zn, and γGT. Excluded variables among females: age, BMI, body fat, Hb, WBC, Plt, IRI, Na, K, Cl, Mg, Zn, and γGT. HDL-C: high-density cholestrerol; HbA1c: hemoglobin A1c; TG: tryglyceride; LDL-C: low-density chokesterol; CRP: C-reactive protein.

## Data Availability

The original contributions presented in this study are included in the article/Supplementary Material. Further inquiries can be directed to the corresponding author.

## Supplementary Materials

The following supporting information can be downloaded at: https://www.mdpi.com/article/10.3390/nu18050813/s1, Figure S1: Box-and-whisker plot of the differences of glucose levels between plasma (Glu(P)) and serum (Glu(S)) among All participants, males, and females; Supplemental Table S1: (A) Simple correlation analysis between glucose concentration and other parameters among all participants; (B) Simple correlation analysis between glucose concentration and other parameters among males; (C) Simple correlation analysis between glucose concentration and other parameters among females; Supplemental Table S2: Parameters for the two groups of positive and negative Glu(P-S) males.

## Abbreviations

The following abbreviations are used in this manuscript:

BMI	Body mass index
Ca	Calcium
Eno1	Enolase 1
Fe	Iron
γGT	Gamma-glutamyltransferase
Glu(P)	Plasma glucose
Glu(P-S)	Difference between plasma glucose and serum glucose
Glu(S)	Serum glucose
Hb	Hemoglobin
HbA1c	Hemoglobin A1c
HDL-C	High-density lipoprotein cholesterol
HOMA-β	Homeostasis Model Assessment of β-Cell Function
HOMA-IR	Homeostasis Model Assessment of Insulin Resistance
IRI_0_	Fasting immunoreactive insulin
LDL-C	Low-density lipoprotein cholesterol
Mg	Magnesium
Na	Sodium
NaF	Sodium fluoride
PG_0_	Fasting plasma glucose
RST	Rapid serum tube
TG	Triglyceride
TyG index	Triglyceride–glucose index
WBC	White blood cell
Zn	Zinc
