# Supplementary material for: A Significantly Higher Glucose Concentration in Plasma Collected with Glycolytic Inhibitors than in Serum: Impact of Insulin Resistance
Source: Nutrients. 2026 Mar 2;18(5):813. doi: 10.3390/nu18050813 (PMC12986600; doi:10.3390/nu18050813)
Supplement: Supplementary file 1 [file nutrients-18-00813-s001.zip › nutrients-4148152-supplementary.pdf]

Data supplements.

Supplemental Figure S1.

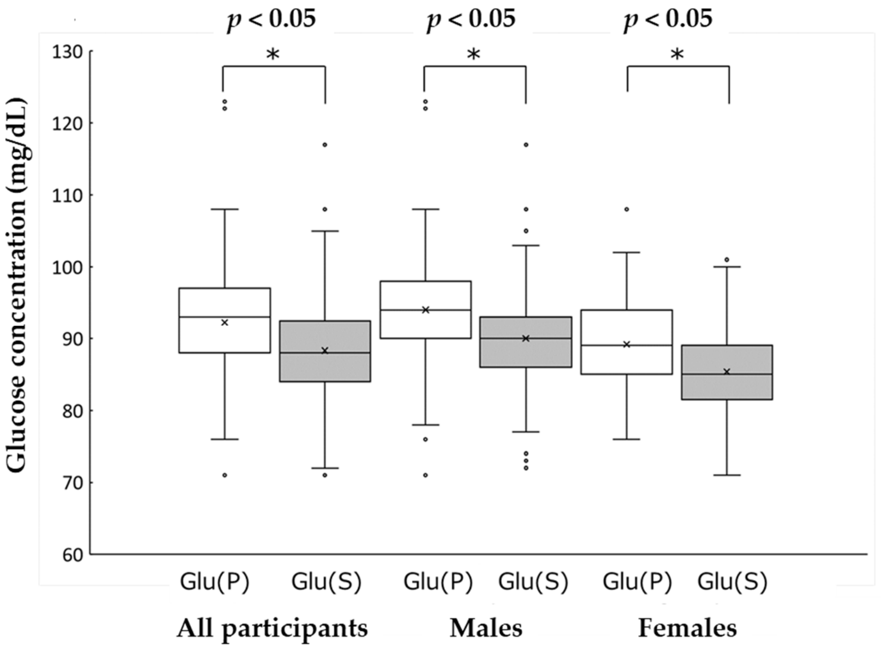

Box-and-whisker plot of the differences of glucose levels between plasma (Glu(P)) and serum (Glu(S)) among All participants (n = 333), males (n = 212), and females (n = 121).
* $p < 0.05$ by Wilcoxon signed rank test with paired samples.

Supplemental Table S1A. Simple correlation analysis between glucose concentration and other parameters among all participants

n=333		Glu(P)	Glu(S)	HbA1c	IRI	HOMA-IR	HOMA-β	HDL-C	TG	LDL-C	TyG-index	RBC	Hb	WBC	Ferritin	Fe	Na	Mg	Zn
BMI	*r*	0.252	0.206	-0.063	0.171	0.196	0.074	-0.358	0.194	0.219	0.218	0.382	0.391	0.119	0.558	0.077	0.1	0.067	0.229
	p	<.001	<.001	0.256	0.002	<.001	0.179	<.001	<.001	<.001	<.001	<.001	<.001	0.031	<.001	0.162	0.07	0.247	0.025
Glu(P)	*r*		0.938	0.079	0.403	0.517	-0.135	-0.235	0.143	0.076	0.284	0.283	0.302	0.096	0.335	0.025	0.064	0.069	0.105
	p		<.001	0.148	<.001	<.001	0.014	<.001	0.009	0.167	<.001	<.001	<.001	0.081	<.001	0.649	0.242	0.232	0.309
Glu(S)	*r*			0.133	0.428	0.535	-0.083	-0.209	0.107	0.078	0.242	0.262	0.274	0.046	0.295	-0.002	0.023	0.034	0.02
	p			0.015	<.001	<.001	0.132	<.001	0.052	0.157	<.001	<.001	<.001	0.402	<.001	0.967	0.676	0.552	0.844
HbA1c	*r*				0	0.012	-0.032	0.073	0.014	0.12	0.025	-0.051	-0.174	0.003	-0.17	-0.225	-0.179	0.115	0.19
	p				0.997	0.824	0.566	0.182	0.797	0.028	0.653	0.358	0.001	0.963	0.026	<.001	0.001	0.045	0.064
IRI	*r*					0.989	0.806	-0.153	0.269	0.19	0.321	0.228	0.214	0.194	0.08	0.068	-0.004	-0.053	-0.006
	p					<.001	<.001	0.005	<.001	<.001	<.001	<.001	<.001	<.001	0.3	0.216	0.942	0.357	0.951
HOMA-IR	*r*						0.721	-0.178	0.275	0.19	0.345	0.252	0.242	0.191	0.128	0.067	0.003	-0.037	0.006
	p						<.001	0.001	<.001	<.001	<.001	<.001	<.001	<.001	0.093	0.225	0.962	0.526	0.951
HOMA-β	*r*							-0.042	0.214	0.173	0.181	0.091	0.058	0.154	-0.124	0.051	-0.057	-0.091	-0.089
	p							0.45	<.001	0.002	<.001	0.098	0.288	0.005	0.105	0.355	0.3	0.114	0.387
HDL-C	*r*								-0.315	-0.03	-0.336	-0.394	-0.356	-0.054	-0.479	-0.098	-0.205	-0.03	-0.179
	p								<.001	0.582	<.001	<.001	<.001	0.327	<.001	0.074	<.001	0.601	0.081
TG	*r*									0.32	0.986	0.321	0.312	0.205	0.152	0.124	0.072	0.062	0.185
	p									<.001	<.001	<.001	<.001	<.001	0.047	0.024	0.19	0.281	0.072
LDL-C	*r*										0.323	0.218	0.168	0.066	0.158	0.076	-0.021	0.033	0.179
	p										<.001	<.001	0.002	0.232	0.039	0.166	0.708	0.569	0.08
TyG index	*r*											0.349	0.347	0.209	0.186	0.121	0.077	0.075	0.204
	p											<.001	<.001	<.001	0.015	0.027	0.16	0.194	0.046
RBC	*r*												0.883	0.046	0.567	0.191	0.173	0.159	0.334
	p												<.001	0.401	<.001	<.001	0.002	0.006	<.001
Hb	*r*													0.041	0.646	0.266	0.185	0.15	0.349
	p													0.454	<.001	<.001	<.001	0.009	<.001
WBC	*r*														-0.043	0.04	-0.005	0.036	-0.039
	p														0.577	0.465	0.933	0.534	0.706
Ferritin	*r*															0.148	0.328	-0.018	0.297
	p															0.053	<.001	0.832	0.003
Fe	*r*																-0.014	0.019	0.128
	p																0.801	0.748	0.214
Na	*r*																	0.094	-0.004
	p																	0.103	0.973
Mg	*r*																		0.075
	p																		0.549

Data are shown as Spearman's rank correlation coefficient for each parameter. BMI: Body Mass Index; HbA1c: hemoglobin A1c; Glu(P): plasma glucose; Glu(S): serum glucose; IRI: immunoreactive insulin; HOMA-IR: Homeostatic Model Assessment for Insulin Resistance; HOMA-β: Homeostatic Model Assessment of beta cell function; HDL-C: high-density lipoprotein cholesterol; TG: triglyceride; LDL-C: low-density lipoprotein cholesterol; TyG index: Triglyceride Glucose index; γGT: γ-glutamyltransferase; Hb: hemoglobin; WBC: white blood cell count; Plt: platelet count; Na: sodium; K: potassium; Cl: chlorine; IP: inorganic phosphorus; Ca: calcium; Mg: magnesium; Fe: iron; Zn: zinc; CRP: C-reactive protein. Statistical significance was set at a p-value of < 0.05. *: $p < 0.05$. Positive correlations are in bold red, negative correlations are in bold blue.

Supplemental Table S1B. Simple correlation analysis between glucose concentration and other parameters among males

n=212		Glu(P)	Glu(S)	HbA1c	IRI	HOMA-IR	HOMA-β	HDL-C	TG	LDL-C	TyG-index	RBC	Hb	WBC	Ferritin	Fe	Na	Mg	Zn
BMI	*r*	0.119	0.075	0.015	**0.204**	**0.206**	**0.209**	**-0.292**	**0.145**	**0.284**	**0.147**	**0.227**	**0.211**	**0.185**	**0.229**	0.065	-0.051	0	0.219
	p	0.085	0.28	0.825	**0.003**	**0.003**	**0.002**	<.001	**0.035**	<.001	**0.032**	<.001	**0.002**	**0.007**	**0.02**	0.349	0.461	0.999	0.119
Glu(P)	*r*		**0.925**	0.134	**0.394**	**0.502**	-0.073	-0.036	0.048	0.076	**0.179**	0.025	0.088	**0.15**	-0.04	0.036	-0.06	0.082	-0.087
	p		<.001	0.051	<.001	<.001	0.292	0.6	0.484	0.27	**0.009**	0.719	0.2	**0.029**	0.689	0.603	0.382	0.254	0.54
Glu(S)	*r*			**0.205**	**0.402**	**0.503**	-0.038	-0.032	-0.006	0.081	0.118	0.024	0.066	0.105	-0.037	-0.01	-0.094	0.047	-0.151
	p			**0.003**	<.001	<.001	0.585	0.642	0.933	0.239	0.087	0.731	0.338	0.127	0.712	0.885	0.173	0.517	0.285
HbA1c	*r*				0.004	0.022	-0.061	0.002	0.014	**0.14**	0.03	0.043	-0.166	-0.029	**-0.248**	-0.126	-0.207	0.067	0.24
	p				0.956	0.753	0.374	0.973	0.835	**0.041**	0.664	0.534	0.016	0.673	**0.011**	0.066	0.002	0.355	0.087
IRI	*r*					**0.99**	**0.849**	**-0.218**	**0.251**	**0.251**	**0.304**	**0.263**	**0.3**	**0.185**	0.032	0.092	0.02	0.002	-0.096
	p					<.001	<.001	**0.001**	<.001	<.001	<.001	<.001	<.001	**0.007**	0.751	0.183	0.774	0.982	0.498
HOMA-IR	*r*						**0.778**	**-0.206**	**0.242**	**0.255**	**0.311**	**0.248**	**0.289**	**0.194**	0.033	0.094	0.001	0.017	-0.093
	p						<.001	**0.003**	<.001	<.001	<.001	<.001	<.001	**0.005**	0.737	0.173	0.987	0.817	0.51
HOMA-β	*r*							**-0.234**	**0.268**	**0.259**	**0.246**	**0.283**	**0.294**	0.106	0.061	0.088	0.064	-0.036	-0.067
	p							<.001	<.001	<.001	<.001	<.001	<.001	0.124	0.541	0.201	0.354	0.622	0.635
HDL-C	*r*								**-0.27**	-0.046	**-0.267**	**-0.168**	-0.061	-0.101	**-0.241**	-0.006	-0.073	-0.043	0.024
	p								<.001	0.502	<.001	**0.014**	0.379	0.143	**0.014**	0.935	0.292	0.551	0.864
TG	*r*									**0.367**	**0.987**	**0.212**	**0.232**	**0.182**	-0.017	**0.138**	-0.013	0.067	0.232
	p									<.001	<.001	**0.002**	<.001	**0.008**	0.863	**0.045**	0.85	0.352	0.098
LDL-C	*r*										**0.371**	**0.3**	**0.266**	0.101	0.158	0.098	-0.054	0.041	0.133
	p										<.001	<.001	<.001	0.145	0.108	0.157	0.43	0.569	0.345
TyG index	*r*											**0.214**	**0.244**	**0.196**	-0.027	**0.14**	-0.018	0.079	0.241
	p											**0.002**	<.001	**0.004**	0.785	**0.042**	0.794	0.27	0.085
RBC	*r*												**0.755**	0.097	-0.042	**0.16**	-0.057	0.112	0.075
	p												<.001	0.158	0.676	**0.02**	0.407	0.12	0.596
Hb	*r*													0.107	0.025	**0.282**	-0.114	**0.17**	0.096
	p													0.12	0.801	<.001	0.097	**0.018**	0.498
WBC	*r*														0.027	0.036	-0.051	0.065	0.014
	p														0.785	0.606	0.456	0.369	0.919
Ferritin	*r*															0.02	0.127	-0.17	-0.025
	p															0.844	0.201	0.117	0.862
Fe	*r*																-0.017	0.04	-0.027
	p																0.804	0.58	0.851
Na	*r*																	-0.054	-0.143
	p																	0.454	0.312
Mg	*r*																		0.139
	p																		0.427

Data are shown as Spearman's rank correlation coefficient for each parameter. BMI: Body Mass Index; HbA1c: hemoglobin A1c; Glu(P): plasma glucose; Glu(S): serum glucose; IRI: immunoreactive insulin; HOMA-IR: Homeostatic Model Assessment for Insulin Resistance; HOMA-β: Homeostatic Model Assessment of beta cell function; HDL-C: high-density lipoprotein cholesterol; TG: triglyceride; LDL-C: low-density lipoprotein cholesterol; TyG index: Triglyceride Glucose index; γGT: γ-glutamyltransferase; Hb: hemoglobin; WBC: white blood cell count; Plt: platelet count; Na: sodium; K: potassium; Cl: chlorine; IP: inorganic phosphorus; Ca: calcium; Mg: magnesium; Fe: iron; Zn: zinc; CRP: C-reactive protein. Statistical significance was set at a p-value of < 0.05. *: $p < 0.05$. Positive correlations are in bold red, negative correlations are in bold blue.

Supplemental Table S1C. Simple correlation analysis between glucose concentration and other parameters among females

		Glu(P)	Glu(S)	HbA1c	IRI	HOMA-IR	HOMA-β	HDL-C	TG	LDL-C	TyG-index	RBC	Hb	WBC	Ferritin	Fe	Na	Mg	Zn
BMI	*r*	0.16	0.109	-0.104	0.089	0.107	0.011	-0.121	0.064	0.124	0.086	-0.113	-0.15	0.13	**0.387**	0.013	0.049	0.109	**-0.305**
	p	0.082	0.237	0.259	0.333	0.247	0.908	0.191	0.493	0.178	0.353	0.221	0.104	0.159	**0.001**	0.892	0.594	0.262	**0.047**
Glu(P)	*r*		**0.909**	0.113	**0.412**	**0.515**	-0.163	-0.13	0.1	0.046	**0.272**	0.027	-0.024	0.032	**0.27**	-0.095	0.015	-0.018	0.148
	p		**<.001**	0.217	**<.001**	**<.001**	0.074	0.156	0.277	0.614	**0.003**	0.773	0.79	0.729	**0.026**	0.301	0.871	0.856	0.336
Glu(S)	*r*			0.145	**0.471**	**0.562**	-0.059	-0.061	0.094	0.046	**0.26**	-0.017	-0.04	-0.018	0.182	-0.101	-0.026	-0.057	0.064
	p			0.112	**<.001**	**<.001**	0.523	0.509	0.304	0.613	**0.004**	0.856	0.663	0.844	0.137	0.271	0.78	0.557	0.677
HbA1c	*r*				0.019	0.023	-0.024	0.042	0.083	0.098	0.109	0.05	-0.097	0.045	-0.026	**-0.353**	-0.091	**0.211**	0.296
	p				0.833	0.806	0.792	0.648	0.366	0.284	0.236	0.586	0.289	0.623	0.836	**<.001**	0.322	**0.028**	0.051
IRI	*r*					**0.99**	**0.785**	0.016	**0.281**	0.071	**0.351**	**0.261**	0.152	0.221	0.082	-0.008	-0.121	-0.175	0.067
	p					**<.001**	**<.001**	0.865	**0.002**	0.439	**<.001**	**0.004**	0.097	0.015	0.506	0.929	0.187	0.07	0.667
HOMA-IR	*r*						**0.704**	-0.014	**0.289**	0.067	**0.376**	**0.242**	0.133	**0.211**	0.123	-0.019	-0.107	-0.16	0.062
	p						**<.001**	0.878	**0.001**	0.466	**<.001**	**0.008**	0.147	**0.02**	0.319	0.836	0.241	0.099	0.689
HOMA-β	*r*							0.146	**0.224**	0.054	**0.19**	**0.279**	**0.179**	**0.207**	-0.048	0.022	**-0.184**	-0.156	0.017
	p							0.111	**0.014**	0.553	**0.037**	**0.002**	**0.049**	**0.023**	0.698	0.811	**0.044**	0.107	0.911
HDL-C	*r*								**-0.225**	0.073	**-0.245**	0.057	0.151	-0.073	-0.052	-0.133	-0.125	0.049	-0.158
	p								**0.013**	0.423	**0.007**	0.532	0.099	0.428	0.671	0.147	0.172	0.616	0.306
TG	*r*									**0.213**	**0.977**	**0.317**	**0.186**	**0.291**	0	0.022	0.062	0.001	-0.092
	p									**0.019**	**<.001**	**<.001**	**0.041**	**0.001**	1	0.813	0.499	0.995	0.553
LDL-C	*r*										**0.213**	0.107	0.03	0.016	-0.031	0.031	-0.03	0.001	0.185
	p										**0.019**	0.241	0.744	0.864	0.8	0.738	0.741	0.992	0.228
TyG index	*r*											**0.319**	**0.184**	**0.286**	0.034	-0.003	0.046	0.001	-0.051
	p											**<.001**	**0.043**	**0.001**	0.785	0.972	0.618	0.992	0.745
RBC	*r*												**0.729**	**0.241**	0.052	0.141	0.028	0.179	0.119
	p												**<.001**	**0.008**	0.674	0.123	0.759	0.064	0.441
Hb	*r*													**0.231**	**0.284**	**0.32**	0.082	0.08	0.19
	p													**0.011**	**0.019**	**<.001**	0.368	0.409	0.216
WBC	*r*														0.152	0.058	0.113	0.001	-0.056
	p														0.216	0.528	0.216	0.992	0.717
Ferritin	*r*															**0.357**	0.039	-0.131	0.18
	p															**0.003**	0.755	0.34	0.243
Fe	*r*																-0.094	-0.035	0.146
	p																0.304	0.716	0.344
Na	*r*																	**0.31**	-0.118
	p																	**0.001**	0.446
Mg	*r*																		-0.074
	p																		0.693

Data are shown as Spearman's rank correlation coefficient for each parameter. BMI: Body Mass Index; HbA1c: hemoglobin A1c; Glu(P): plasma glucose; Glu(S): serum glucose; IRI: immunoreactive insulin; HOMA-IR: Homeostatic Model Assessment for Insulin Resistance; HOMA-β: Homeostatic Model Assessment of beta cell function; HDL-C: high-density lipoprotein cholesterol; TG: triglyceride; LDL-C: low-density lipoprotein cholesterol; TyG index: Triglyceride Glucose index; γGT: γ-glutamyltransferase; Hb: hemoglobin; WBC: white blood cell count; Plt: platelet count; Na: sodium; K: potassium; Cl: chlorine; IP: inorganic phosphorus; Ca: calcium; Mg: magnesium; Fe: iron; Zn: zinc; CRP: C-reactive protein. Statistical significance was set at a p-value of < 0.05. *: $p < 0.05$. Positive correlations are in bold red, negative correlations are in bold blue.

Supplemental Table S2. Parameters for the two groups of positive and negative Glu(P-S) males

	Males (n=212)			Females (n=121)		
	Glu(P) > Glu(S) (n = 197)	Glu(P) < Glu(S) (n = 7)	*p*	Glu(P) > Glu(S) (n = 113)	Glu(P) < Glu(S) (n = 3)	*p*
age (years)	26 (24 - 28)	24 (23 - 25)	0.226	23 (23 - 24)	23 (23 - 24)	0.603
BMI (kg/m^2)	22.5 (20.8 - 24)	22.3 (22.2 - 24.6)	0.29	20.3 (19 - 22)	19.6 (18.7 - 20.4)	0.636
body fat （%）	18.5 (15.6 - 22.8)	19.6 (15.5 - 22.8)	0.85	27.9 (24.7 - 30.4)	24.4 (22 - 25.5)	0.806
Plasma glucose (mg/dL)	94 (90 - 97)	96 (92 - 102)	0.432	89 (86 - 94)	85 (81 - 88)	0.103
Serum glucose (mg/dL)	90 (86 - 93)	98 (93 - 103)	**0.026***	85 (82 - 90)	87 (81 - 88)	0.897
HbA1c (%)	5.4 (5.2 - 5.5)	5.4 (5.2 - 5.5)	0.987	5.4 (5.3 - 5.5)	5.4 (5.3 - 5.6)	0.138
IRI (μU/mL)	6.7 (5.4 - 9.2)	7.1 (5.4 - 9.6)	0.887	6.5 (4.9 - 8.4)	7 (4.9 - 12.2)	0.617
HOMA-IR	1.54 (1.2 - 2.22)	1.75 (1.35 - 2.26)	0.731	1.38 (1.02 - 1.9)	1.52 (0.94 - 2.61)	0.783
HOMA-β	81 (64 - 104)	89 (60 - 128)	0.709	89 (74 - 118)	141 (103 - 188)	0.121
HDL-C (mg/dL)	58 (50 - 65)	59 (51 - 68)	0.853	72 (64 - 79)	78 (62 - 81)	0.569
TG (mg/dL)	65 (51 - 92)	72 (52 - 77)	0.557	50 (41 - 65)	62 (49 - 93)	0.938
LDL-C (mg/dL)	101 (86 - 121)	101 (95 - 118)	0.688	99 (82 - 116)	78 (68 - 127)	0.535
TyG index	8.05 (7.78 - 8.37)	7.95 (7.8 - 8.19)	0.544	7.73 (7.52 - 7.99)	7.76 (7.65 - 8.25)	0.959
γGT (mg/dL)	20 (15 - 26)	15 (13 - 20)	0.05	13 (11 - 18)	14 (11 - 17)	0.993
Hb (g/dL)	15.6 (15.1 - 16.3)	15.6 (15.6 - 16)	0.661	13.6 (12.9 - 14)	13.4 (13.3 - 14.2)	0.24
WBC (×10^3/μL)	4.9 (4.3 - 5.7)	4.5 (4.1 - 5.7)	0.488	5.2 (4.4 - 6)	4.8 (4.4 - 5)	0.463
Plt (×10^3/μL)	230 (206 - 260)	218 (201 - 246)	0.466	258 (225 - 277)	232 (217 - 257)	0.384
Na (mEq/L)	142 (141 - 143)	142 (140 - 143)	0.979	141 (140 - 142)	141 (140 - 143)	0.439
K (mEq/L)	4.1 (3.9 - 4.2)	4.1 (3.9 - 4.2)	0.624	4 (3.8 - 4.1)	4.1 (3.9 - 4.2)	0.774
Cl (mEq/L)	103 (102 - 105)	104 (103 - 105)	0.446	104 (103 - 105)	104 (103 - 106)	0.462
IP (mg/dL)	3.6 (3.2 - 3.8)	3.9 (3.7 - 3.9)	0.15	3.7 (3.5 - 4)	3.7 (3.5 - 4)	0.593
Ca (mg/dL)	9.7 (9.5 - 9.9)	9.6 (9.5 - 9.9)	0.844	9.5 (9.3 - 9.7)	9.8 (9.6 - 9.9)	0.855
Mg (mg/dL)	2.1 (2 - 2.2)	2.1 (2.1 - 2.1)	0.811	2.1 (2 - 2.2)	2 (1.9 - 2.1)	0.125
Fe (mg/dL)	123 (92 - 151)	82 (76 - 111)	**0.038***	111 (83 - 144)	103 (97 - 117)	0.673
ferritin (ng/mL)	136 (86 - 196)	134 (113 - 144)	0.688	31 (17 - 48)	46 (36 - 52)	0.447
Zn (μg/dL)	95 (87 - 101)	88 (81 - 106)	0.738	88 (82 - 94)	85 (81 - 87)	0.467
CRP (mg/dL)	0.02 (0.01 - 0.06)	0.04 (0.04 - 0.14)	0.11	0.02 (0.01 - 0.04)	0.02 (0.01 - 0.03)	0.902

Data are shown as medians (first quartile–third quartile). BMI: Body Mass Index; Glu(P-S): plasma glucose – serum glucose; HbA1c: hemoglobin A1c; IRI: immunoreactive insulin; HDL-C: high-density lipoprotein cholesterol; TG: triglyceride; LDL-C: low-density lipoprotein cholesterol; TyG index: Triglyceride Glucose index; γ GT: γ-glutamyltransferase; Hb: hemoglobin; WBC: white blood cell count; Plt: platelet count; Na: sodium; K: potassium; Cl: chlorine; IP: inorganic phosphorus; Ca: calcium; Mg: magnesium; Fe: iron; Zn: zinc; CRP: C-reactive protein. Significant between-groups differences were identified using the Mann–Whitney U test. Statistical significance was set at a p-value of < 0.05. *: p < 0.05. Glu(P)>Glu(S) values is higher tham Glu(P)<Glu(S): red bold; Glu(P)>Glu(P) value is lower than Glu(P)<Glu(S) value: blue bold.
